# High flow biphasic positive airway pressure by helmet – effects on pressurization, tidal volume, carbon dioxide accumulation and noise exposure

**DOI:** 10.1186/cc7907

**Published:** 2009-06-05

**Authors:** Onnen Moerer, Peter Herrmann, José Hinz, Paolo Severgnini, Edoardo Calderini, Michael Quintel, Paolo Pelosi

**Affiliations:** 1 Department of Anaesthesiology, Emergency and Critical Care Medicine, University of Göttingen, Robert-Koch-Strasse 40, 37075 Göttingen, Germany; 2 Dipartimento di Anestesia, Rianimazione e Terapia del Dolore, Fondazione Ospedale Maggiore Policlinico, Mangiagalli e Regina Elena, IRCCS, via Francesco Sforza 28, 20122 Milano, Italy; 3 Department of Ambient, Health and Safety, c/o Villa Toeplitz Via G.B. Vico, 46, 21100 Varese, Italy

## Abstract

**Introduction:**

Non-invasive ventilation (NIV) with a helmet device is often associated with poor patient-ventilator synchrony and impaired carbon dioxide (CO_2_) removal, which might lead to failure. A possible solution is to use a high free flow system in combination with a time-cycled pressure valve placed into the expiratory circuit (HF-BiPAP). This system would be independent from triggering while providing a high flow to eliminate CO_2_.

**Methods:**

Conventional pressure support ventilation (PSV) and time-cycled biphasic pressure controlled ventilation (BiVent) delivered by an Intensive Care Unit ventilator were compared to HF-BiPAP in an in vitro lung model study. Variables included delta pressures of 5 and 15 cmH2O, respiratory rates of 15 and 30 breaths/min, inspiratory efforts (respiratory drive) of 2.5 and 10 cmH2O) and different lung characteristics. Additionally, CO_2 _removal and noise exposure were measured.

**Results:**

Pressurization during inspiration was more effective with pressure controlled modes compared to PSV (*P *< 0.001) at similar tidal volumes. During the expiratory phase, BiVent and HF-BiPAP led to an increase in pressure burden compared to PSV. This was especially true at higher upper pressures (*P *< 0.001). At high level of asynchrony both HF-BiPAP and BiVent were less effective. Only HF-BiPAP ventilation effectively removed CO2 (*P *< 0.001) during all settings. Noise exposure was higher during HF-BiPAP (*P *< 0.001).

**Conclusions:**

This study demonstrates that in a lung model, the efficiency of NIV by helmet can be improved by using HF-BiPAP. However, it imposes a higher pressure during the expiratory phase. CO2 was almost completely removed with HF-BiPAP during all settings.

## Introduction

Non-invasive ventilation (NIV) has been increasingly used in intensive care patients [1-7]. Problems with the commonly used interfaces of the NIV application include air leakage [8,9], patient discomfort [10], and pressure-related ulcerations of the nose [11]. All of these problems can limit the duration of NIV and account for failures [12]. Navalesi and colleagues [9] demonstrated that interface design in NIV is important with regard to a patient's tolerance and the time that NIV can be applied.

A new NIV interface, the helmet, has been tested in different clinical situations [13-16]. The helmet is associated with a better tolerance and a lower rate of interface-associated complications [14]. However, due to the large collapsible and compliant chamber that encompasses the patient's head, the helmet impairs patient-ventilator synchrony with conventional pneumatic systems [17,18]. Furthermore, it reduces the work of breathing less effectively than conventional facial masks do [17,19].

A further problem with the helmet is related to the insufficient removal of carbon dioxide (CO_2_). This issue is especially problematic during positive pressure ventilation (PSV) [19,20]. The helmet may impair gas exchange and increase the work of breathing. In addition, increases in water vapour with low flows [21] and temperature may increase discomfort. To overcome these problems, we recently developed a specially designed system with a high free flow source connected to the inspiratory limb of the helmet. The device has a time-cycled valve positioned on the expiratory limb. The valve provides biphasic positive airway pressure (HF-BiPAP). This device is easy to handle and provides two different pressure levels. Overall, it might improve patient comfort and maximize CO_2 _washout.

This study compared HF-BiPAP with PSV and biphasic positive airway pressure (BiVent). These modalities were delivered by a high performance conventional ventilator using the helmet as an interface. The study was performed using a lung model capable of spontaneous breathing. The model mimicked normal, restrictive, and obstructive respiratory patterns.

## Materials and methods

### Equipment and setup

#### NIV interface

**Figure 1:**
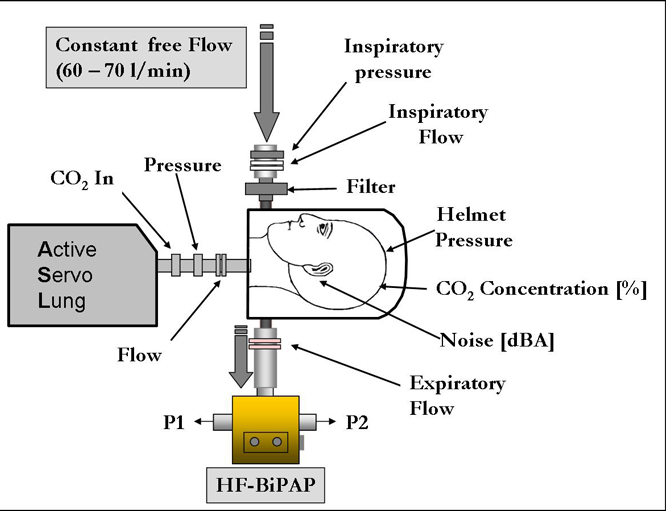
Setup for the study of the HF-BiPAP system. The inspiratory tube was directly connected to the hospital's gas supply via a flow meter, while the expiratory tube was connected to the HF-BiPAP. During conventional PSV and BiVent, the inspiratory and expiratory tubes were connected to the ventilator. BiVent = time-cycled pressure controlled switching between two continuous positive airway pressure levels; HF-BiPAP = high flow biphasic positive airway pressure; PSV = pressure support ventilation.

Measurements were performed with a helmet (4Vent, Rüsch, Medical GmbH, Kernen, Germany) placed on a mannequin head (Airway Management Trainer, Laerdal Medical, Stavanger, Norway) connected to a breathing simulator (ASL 5000™, Ingmar Medical Ltd., Pittsburgh, PA, USA; Figure 1). Two underarm laces attached to a ring at the lower side of the helmet prevented it from lifting when inflated. A plastic collar, fitted around the neck, prevented leakage during ventilation. Inspiratory and expiratory tube connectors were fitted to the lower part of the helmet.

#### Modes of ventilation and ventilator tested

We compared PSV and BiVent delivered by a conventional high-performance mechanical ventilator (Servo-i Maquet Critical Care AB, Solna, Sweden) with the new HF-BiPAP.

PSV was applied in NIV mode with the steepest rise time and the cycle off at 25% of peak inspiratory flow. In the NIV-mode, trigger sensitivity is adjusted automatically.

BiVent was performed with a time-cycled switch between the two continuous positive airway pressure (CPAP) levels. This setting is comparable with BiPAP/airway pressure release ventilation. The steepest rise time was chosen and no supplementary pressure support of the spontaneous breaths was applied.

HF-BiPAP was performed using a free continuous flow (air and/or oxygen) system delivered by a Venturi system (or other gas delivery systems) connected to the inspiratory limb of the helmet. There was a dedicated device (BiPulse Ventilator, DIMAR, Mirandola, Italy) with a pneumatic time-cycled expiratory valve that was able to transform classic free continuous flow CPAP techniques into biphasic positive airway pressure (Figure 1). The device is composed of a rotating pneumatic valve, two pneumatic timers, and one pneumatic interrupter. The rotating pneumatic time-cycled valve alternates flow between the two outlets. Its geometrical spherical shape makes it impossible for the valves to close completely, even in the absence of an external pneumatic energy supply. Even if the valve is blocked, the sum of the two areas for flow delivery around the spherical valve is equal to the full area in each position (1/2 + 1/2 = 1, 1/3 + 2/3 = 1 etc). The pneumatic interrupter is activated by a time-cycled increase in pneumatic pressure. This pressure is delivered by compressed air/oxygen from the wall or external tank and does not require electrical power. The pneumatic interrupter modulates the pressure on a thin membrane by means of a 'pin valve', which is able to modify the valve's position: the higher the diameter of the pin valve, the less time needed to activate the valve (and vice versa). The pneumatic valve's flow area is 255 mm^2^. The auto-positive end-expiratory pressure (PEEP) generated by the valve is directly proportional to the flow passing throughout the system. Therefore, small flow adjustments were necessary in order to reach the target PEEP. The PEEP was generated by a specific Automatic Pressure Limited valve (DIMAR, Mirandola, Italy), which can be externally regulated by modification of the internal lumen's flow resistance.

#### Lung model

We used a lung model capable of simulating spontaneous breathing (ASL 5000™, Ingmar Medical Ltd., Pittsburgh, PA, USA). This active servo lung consisted of an electrically driven pneumatic lung simulator that allowed for adjustment of the tidal volume, respiratory rate, compliance, resistance, inspiratory effort, inspiratory to expiratory ratio, and the pattern of the inspiration (e.g. rise time and plateau). During the study, data were gathered by sensors placed in the respiratory circuit (Figure 1, described below), not by the lung model.

### Study protocol

#### Ventilatory settings

The respiratory rate during PSV followed the rate set by the lung model. In the case of BiVent and HF-BiPAP, it was fixed on the device at 15 and 30 breaths per minute. For both controlled modes of ventilation an inspiratory:expiratory ratio of 1:1 was chosen.

An additional setting with BiVent and HF-BiPAP cycled at a rate of 15 breaths per minute, while the lung model at a respiratory rate of 30 breaths per minute was measured to simulate extreme asynchrony during the time cycled ventilator modes.

The lower pressure level (P1) was kept constant at a target of 8 cmH_2_O. The Δ pressure above P1 was set to 5 and 15 cmH_2_O. There was no free adjustable flow in PSV/BiVent. For HF-BiPAP the flow was set at about 60 l/minute.

#### Lung model setting

We tested the following conditions: normal lung (normal compliance of 90 ml/cmH_2_O and resistance of 3 cmH_2_O/l); restrictive lung (low compliance of 30 ml/cmH_2_O, normal resistance of 3 cmH_2_O/l); obstructive lung (normal compliance of 90 ml/cmH_2_O, high resistance of 15 cmH_2_O/l).

Measurements were performed at two different inspiratory efforts (low: 2.5 and high: 10 mbar) at a respiratory rate of 15 and 30 breaths per minute. CO_2 _was inflated at 200 ml/minute.

### Measurements

#### Respiratory mechanics

The ventilator was connected by standard disposable ventilator tubes (B&P Beatmungsprodukte GmbH; Neunkirchen, Germany). Gas flow was measured with a pneumotachometer (Fleisch II; Fleisch; Lausanne, Switzerland) connected to the inspiratory side of the helmet (Figure 1). The signals were integrated to obtain volume during off-line evaluation. The pneumotachometer was calibrated by a mass flow meter (TSI 4040 D; TSI Inc.; Shoreview, MN, USA).

Airway pressure was measured at the inspiratory side before the helmet and at the level of the trachea with differential pressure transducers (Sensortechnics; Puchheim, Germany). The transducers were adjusted meticulously at zero flow before each measurement. Additionally the start and end points of inspiration were transferred from the lung model via a digital output (5V TTL – signal) in order to synchronize the data. All signals were sampled at a sampling rate of 100 Hz and digitised via an analogue digital converter (NI-USB 6008, National Instruments, Austin, TX, USA) with a full 12-bit resolution when sampling multiple channels. The acquired signals were displayed and stored online on a standard personal computer using custom-made data acquisition software (BreathAssist V.1.02) programmed LabVIEW™ (National Instruments, Austin, TX, USA).

#### Carbon dioxide measurements

Measurements of CO_2 _removal were performed separately. CO_2 _was injected into the lung at 200 ml/minute via a side port connected to the lung (Figure 1). The resulting CO_2 _concentration within the helmet was measured continuously (CS/3, Datex-Engström, Helsinki, Finland) (Figure 1). The CO_2 _concentrations for each setting were acquired during steady-state conditions after a wash-in phase [20].

#### Noise exposure

Noise measurements were performed separately. Prior to testing each setting (e.g. lung condition and respiratory rate) we measured a baseline noise level with and without activation of the lung simulator.

Noise exposure was evaluated by a sound level meter (SE 322, Voltcraft, Conrad, Electronics, Hirschau, Germany) placed within the helmet near the mannequin's ear. The sensor acquired the noise level at a sampling rate of 10 Hz. Measurements were transferred online to a personal computer via a serial interface.

### Data analysis

**Figure 2:**
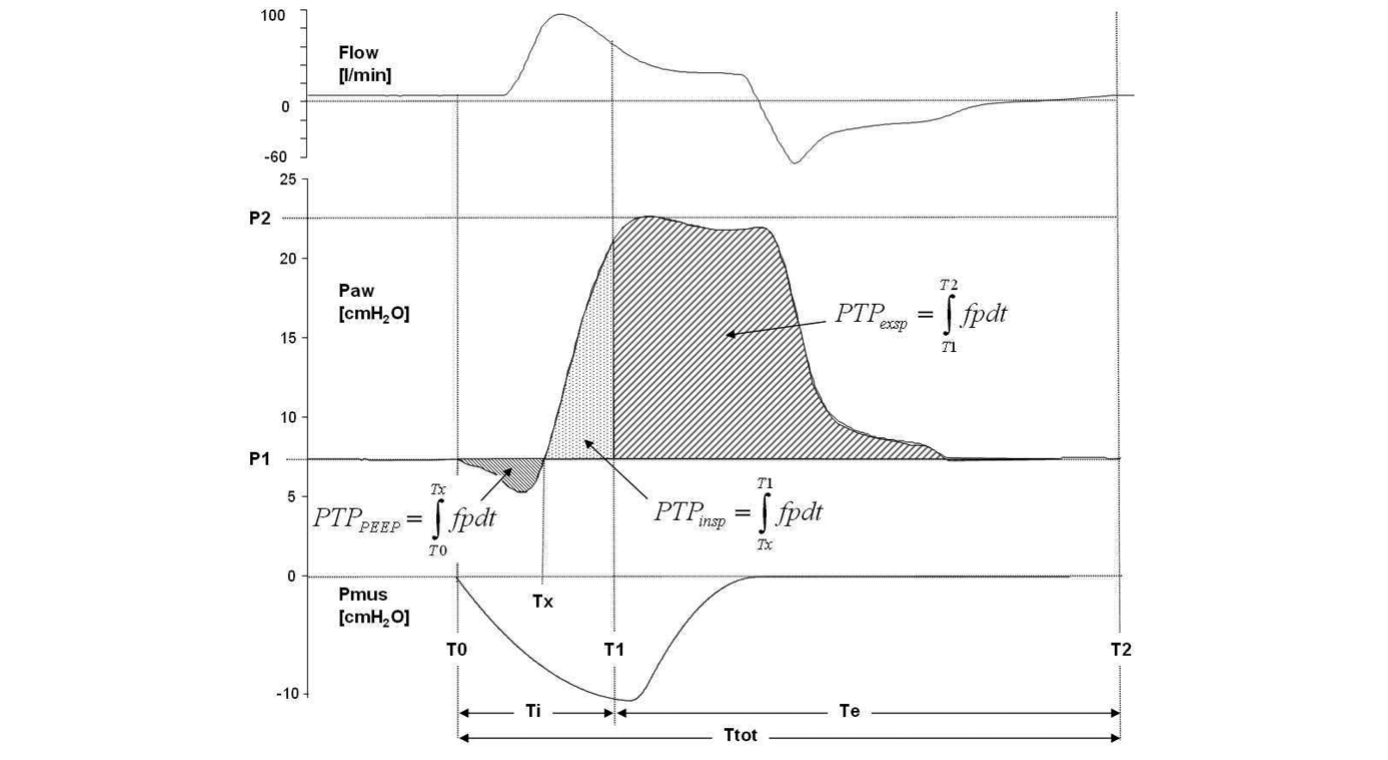
Helmet flow (l/min), helmet pressure (Paw) and muscle pressure (Pmus) tracings during helmet non-invasive ventilation. Td (Trigger delay) indicates the time between the onset of Pmus (T_0_) and the onset of ventilator assistance. Pressure time products (PTP) were calculated from the time between the onset of inspiration and the pressure below PEEP (PTP_PEEP_), the pressure above positive end-expiratory end pressure (PEEP) from onset to the end of inspiration (PTPinsp) as well as for the expiratory phase (PTP_exp_). Note: As the lower pressure represents the target pressure, PTPexp was calculated as the pressure time product beyond PEEP/P1 in order to calculate the extra pressure imposed due to poor synchronization. Tracings were measured during pressure support ventilation (normal lung, respiratory rate 30 bpm, Δ pressure 15 cmH_2_O).

The actual lower, upper, and mean pressures and tidal volumes within the helmet were calculated at all settings. Additionally, the airway pressures and tidal volumes delivered to the lung were calculated, as well as the following pressure time products (PTP) throughout the respiratory cycle based on the inspiratory signal of the active lung (Figure 2): PTP_PEEP_, which is the PTP caused by a pressure drop below PEEP/P1 during inspiration; PTPinsp, which is the PTP above PEEP/P1 during the inspiratory phase; and PTPexp, which is the PTP above PEEP/P1 during the expiratory phase.

Maximum and minimum CO_2 _concentrations as well as peak, minimum, and mean noise exposures were measured separately during all conditions. All data was gathered and analyzed using custom-made software programmed with LabVIEW™ (National Instruments, Austin, TX, USA). Commercially available software was also used (Statistica 8.0, Statsoft, Inc., and Microsoft Excel).

### Statistical analysis

For all conditions, 15 measurements were obtained. The data was presented as mean ± standard deviation (with median and 25th and 75th percentiles when necessary). A multivariate analysis (Wilks' Lambda test of multivariate independence) was performed to detect significant differences between the different experimental conditions. For measurements with high discrepancy between cycling rate (rate of 15 breaths per minute) and respiratory rate (rate of 30 breaths per minute) during HF-BiPAP and BiVent Friedman-analysis of variance (ANOVA) and Wilcoxon tests were used as well as Kruskal-Wallis-ANOVA for the analysis of medians were performed. A *P *value less than or equal to 0.05 was considered to be significant.

## Results

**Figure 3:**
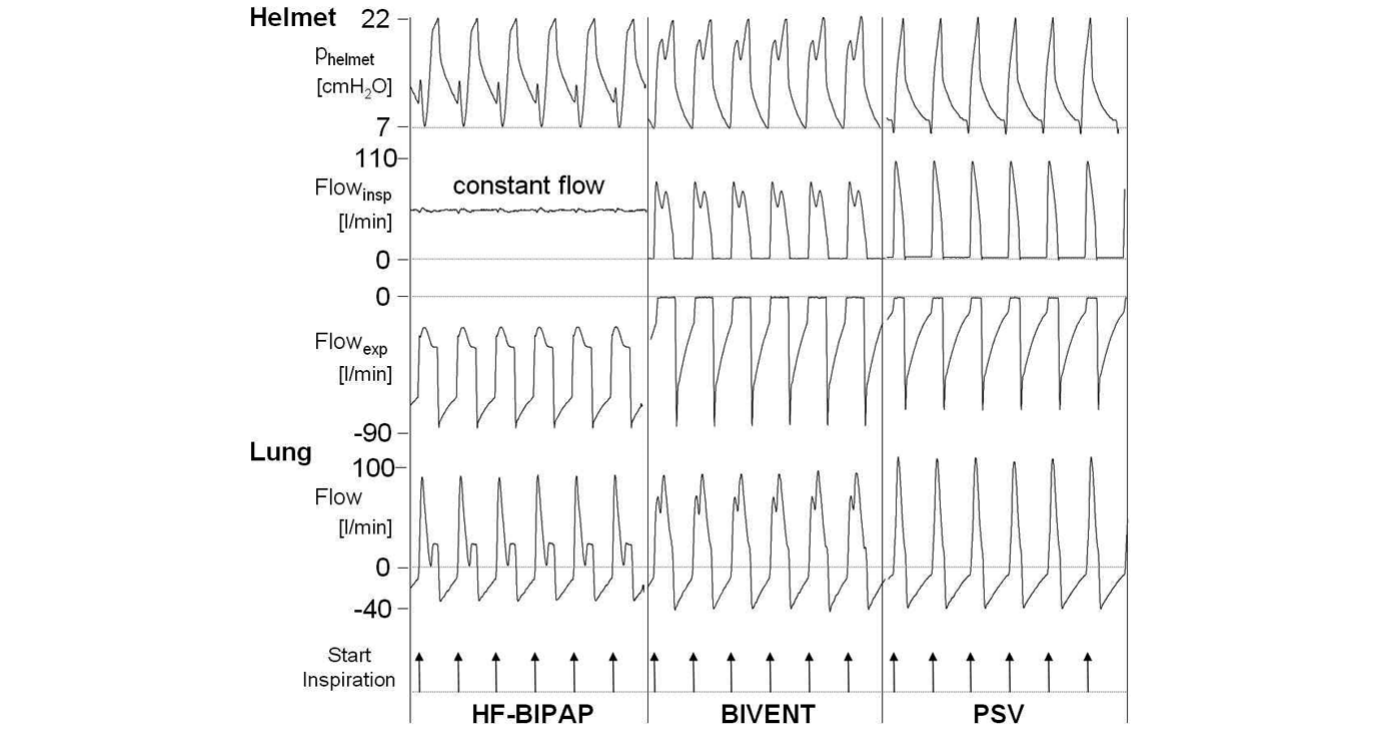
Original tracing of helmet and airway (lung) flow and pressure during HF-BiPAP, BIVENT, and PSV. Compliance was 90 ml/cm H_2_O, resistance was 3 cm H_2_O/l/s, at high inspiratory effort, respiratory rate was 30 breaths per minute and delta pressure was 15 cmH_2_O. Note: During HF-BiPAP inspiratory flow to the helmet was constantly high at about 60 l/minute. Expiratory flow did not become zero due to the high constant free flow. BiVent = time-cycled pressure controlled switching between two continuous positive airway pressure levels; HF-BiPAP = high flow biphasic positive airway pressure; PSV = pressure support ventilation.

Figure 3 shows an original tracing of flow and pressure during HF-BIPAP, BIVENT, and PSV at the helmet and airway level. Pressurization differed due to fixed inspiratory timing. During HF-BiPAP, there was a constant free inspiratory flow between 60 and 70 L/minute. Overall, the mean lower pressures (PEEP/P1) were 8.3 ± 0.4 cmH_2_O (PSV), 8.3 ± 0.6 cmH_2_O (BiVent), and 8.4 ± 0.7 cmH_2_O (HF-BiPAP; *P *= 0.26). There was no significant difference between the tested modes regarding the mean Δ pressure at low (PSV: 5.3 ± 0.4 cmH_2_O, BiVent: 5.4 ± 0.6 cmH_2_O, HF-BiPAP: 5.3 ± 0.9 cmH_2_O; *P *= 0.119) and high upper pressure (PSV: 15.2 ± 0.7 cmH_2_O, BiVent: 15 ± 1 cmH_2_O, HF-BiPAP; 15.1 ± 2.4 cmH_2_O; *P *= 0.308).

### Airway pressures and pressure time products

Although helmet and airway pressure significantly differed (*P *< 0.001), the difference was small. Therefore only airway pressures were reported. The mean airway pressure was influenced by the Δ pressure (*P *< 0.001) and the respiratory rate (*P *< 0.001). Lung conditions had no effect on mean airway pressure (*P *= 0.336). During HF-BiPAP (12.6 ± 2.2 cmH_2_O) and BiVent (12.6 ± 2.7 cmH_2_O) mean airway pressure was higher compared with the PSV setting (10.6 ± 1.8 cmH_2_O; *P *< 0.001; Table 1).

**Table 1:** Mean inspiratory airway pressure (Paw mean), expiratory pressure time product (PTPexp), and maximum CO_2 _concentration (CO_2 _max) within the helmet at a respiratory rate of 15 and 30 breaths per minute

**Normal lung condition**									
**Respiratory rate**		**15 **breaths/minute				**30 **breaths/minute			
**Δ pressure**		**5 **cmH_2_0		**15 **cmH_2_0		**5 **cmH_2_0		**15 **cmH_2_0	
**Effort**		low	high	low	high	low	high	low	high
**Paw mean **(cmH_2_O)	HF-BiPAP	10.2 ± 0.3	10.4 ± 0.4	15.2 ± 0.4	14.7 ± 0.3	10.7 ± 0.2	11.1 ± 0.5	15.1 ± 0.5	15.0 ± 0.2
	BiVent	10.1 ± 0.1	9.8 ± 0.1	14.8 ± 0.3	14.5 ± 0.1	10.6 ± 0.2	9.8 ± 0.2	15.0 ± 0.3	15.2 ± 0.3
	PSV	8.7 ± 0.2	8.4 ± 0.2	11.9 ± 0.6	11.6 ± 0.7	9.6 ± 0.4	8.7 ± 0.2	12 ± 0.4	13.2 ± 0.9

**PTPexp**. (cmH_2_0/sec)	HF-BiPAP	4.3 ± 1.1	10.6 ± 3.2	13.9 ± 1.1	21.4 ± 1.7	3.1 ± 0.4	7.4 ± 1.4	2.7 ± 0.7	9.2 ± 1.2
	BiVent	4.6 ± 0.5	5.7 ± 0.3	18.7 ± 0.7	21.7 ± 0.8	2.8 ± 0.2	2.9 ± 0.8	9.8 ± 0.6	9.4 ± 0.8
	PSV	1.9 ± 0.5	3.2 ± 0.7	6.7 ± 0.4	7 ± 0.6	1.9 ± 0.9	2.5 ± 0.3	4.4 ± 4.9	2.2 ± 0.7

**CO_2 _max **(%)	HF-BiPAP	0.1 ± 0	0.1 ± 0	0.1 ± 0	0.1 ± 0	0.1 ± 0	0.1 ± 0	0.2 ± 0	0.2 ± 0
	BiVent	4.2 ± 0	4.0 ± 0.1	2.1 ± 0	3.2 ± 0	3.9 ± 0.1	2.6 ± 0	1.9 ± 0	1.4 ± 0.2
	PSV	0.2 ± 0	1.2 ± 0	1 ± 0	0.8 ± 0	0.9 ± 0	0.7 ± 0	1 ± 0.2	0.5 ± 0

**Obstructive lung condition**									
**Respiratory rate**		**15 **breaths/minute				**30 **breaths/minute			
**Δ pressure**		**5 **cmH_2_0		**15 **cmH_2_0		**5 **cmH_2_0		**15 **cmH_2_0	
**Effort**		low	high	low	high	low	high	low	high

**Paw mean **(cmH_2_O)	HF-BiPAP	10.4 ± 0.1	9.9 ± 0.1	14.1 ± 0.1	14 ± 0.1	10.2 ± 0.1	10.7 ± 0	14.6 ± 0.1	15.6 ± 0.2
	BiVent	9.9 ± 0.2	10.1 ± 0.1	15.5 ± 0.1	14.9 ± 0	9.9 ± 0.1	10.3 ± 0	15.3 ± 0.2	15.8 ± 0.2
	PSV	9.3 ± 0.1	9.1 ± 0	11.6 ± 0.1	10.6 ± 0.1	9.1 ± 0.1	9.3 ± 0.1	11.2 ± 0.5	12.3 ± 0.1

**PTPexp**. (cmH_2_0/sec)	HF-BiPAP	9 ± 0.5	3.4 ± 0.6	15.9 ± 1.1	19.4 ± 0.9	7.7 ± 0.9	5.7 ± 0.8	11.1 ± 2.3	16.9 ± 3.5
	BiVent	6 ± 0.5	7.4 ± 0.6	18.5 ± 0.7	19.5 ± 0.3	5.9 ± 0.4	6.1 ± 0.1	9.4 ± 0.3	8 ± 0.4
	PSV	3.7 ± 0.7	2.4 ± 0.3	3.2 ± 0.9	2.2 ± 0.3	3.8 ± 0.4	5.5 ± 0.3	2.5 ± 2	4 ± 0.3

**CO_2 _max **(%)	HF-BiPAP	0.1 ± 0	0.1 ± 0	0.2 ± 0	0.2 ± 0	0.1 ± 0	0.1 ± 0	0.2 ± 0	0.2 ± 0
	BiVent	2.5 ± 0	3 ± 0.1	2.8 ± 0	3.2 ± 0	4.8 ± 0	4.8 ± 0	2.3 ± 0	2.2 ± 0
	PSV	0.2 ± 0	0.9 ± 0	0.1 ± 0	0.6 ± 0	0.4 ± 0	0.4 ± 0	0.1 ± 0	0.2 ± 0

**Restrictive lung condition**									
**Respiratory rate**		**15 **breaths/minute				**30 **breaths/minute			
**Δ pressure**		**5 **cmH_2_0		**15 **cmH_2_0		**5 **cmH_2_0		**15 **cmH_2_0	
**Effort**		low	high	low	high	Low	high	low	high

**Paw mean **(cmH_2_O)	HF-BiPAP	9.9 ± 0.3	10.7 ± 0.3	14.6 ± 0.2	14.3 ± 0.3	10.1 ± 0.2	11.5 ± 0.3	14.4 ± 0.1	14.9 ± 0.1
	BiVent	9.9 ± 0.2	9.9 ± 0.1	14.9 ± 0.1	14.9 ± 0.2	10.0 ± 0.3	9.8 ± 0.1	16.3 ± 0.2	16.3 ± 0.2
	PSV	11.6 ± 0.6	9.3 ± 0.1	10.1 ± 0.1	9.8 ± 0.1	10.2 ± 0.3	9.1 ± 0	15.9 ± 0.4	11.3 ± 0.1

**PTP exp**. (cmH_2_0/sec)	HF-BiPAP	6.4 ± 2.9	10.1 ± 3	13.8 ± 1.5	15.0 ± 9.6	3.3 ± 1	4.8 ± 1.1	9.5 ± 2.4	11.7 ± 1.3
	BiVent	5.4 ± 1.7	6.6 ± 1	9.0 ± 0.6	12.1 ± 0.6	3.0 ± 0.3	2.5 ± 0.3	6.6 ± 0.5	8.9 ± 0.7
	PSV	5.2 ± 0.5	4.5 ± 0.7	3.1 ± 0.4	3.3 ± 0.4	3.0 ± 0.8	2.5 ± 0.3	5.6 ± 0.8	4.9 ± 0.6

**CO_2 _max **(%)	HF-BiPAP	0.1 ± 0	0.1 ± 0	0.2 ± 0	0.2 ± 0	0.1 ± 0	0.1 ± 0	0.1 ± 0	0.3 ± 0
	BiVent	4.7 ± 0	4.7 ± 0.1	4.2 ± 0	4.4 ± 0.1	4.1 ± 0	3.0 ± 0.1	3.8 ± 0	2.9 ± 0
	PSV	0.4 ± 0	0.8 ± 0	0.3 ± 0	0.4 ± 0	0.4 ± 0	0.7 ± 0	1.0 ± 0.1	0.6 ± 0

Measurements were made in normal (compliance 90 ml/cmH_2_O, resistance 3 cmH_2_O/l/s), restrictive (compliance 30 ml/cmH_2_O, resistance 3 cmH_2_O/l/s) and obstructive (compliance 90 ml/cmH_2_O, resistance 15 cmH_2_O/l/s) lung conditions, during varying delta pressure (5 and 15 cmH_2_O) and inspiratory efforts (low: 2.5 cmH_2_O, high: 10 cmH_2_O) at a set positive end-expiratory pressure of 8 cmH_2_O.

HF-BiPAP = high flow biphasic positive airway pressure ventilation; BiVent = biphasic positive airway pressure delivered by a ventilator; PSV = pressure support ventilation delivered by a ventilator.

**Figure 4:**
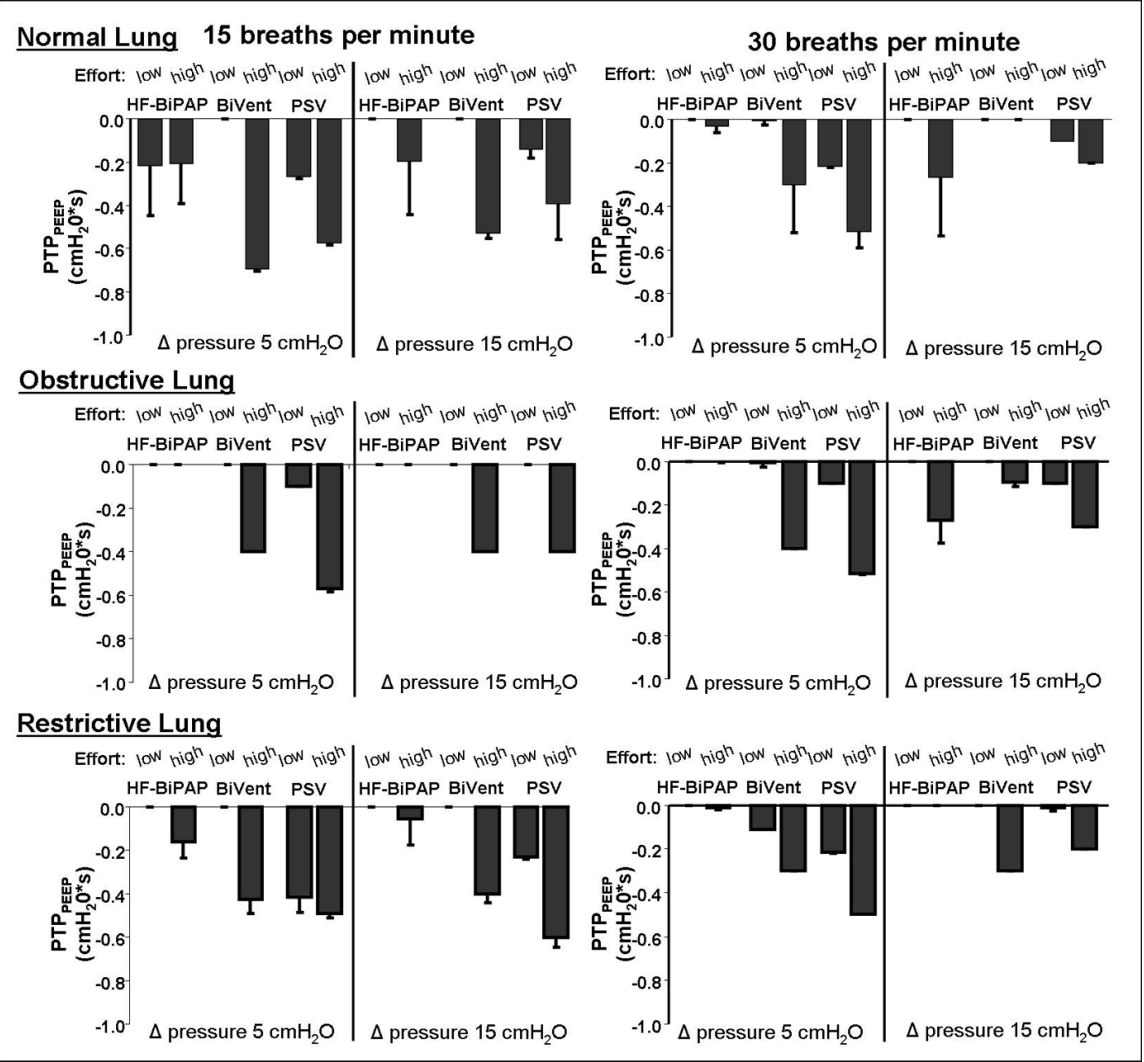
Effect of different ventilator respiratory settings on the mean airway pressure time product below PEEP (PTP_PEEP_) during HF-BiPAP, BiVent, and PSV ventilation. Data were measured in normal (compliance 90 ml/cmH_2_O, resistance 3 cm H_2_O/l/s), restrictive (compliance 30 ml/cmH_2_O, resistance 3 cmH_2_O/l/s), and obstructive lung conditions (compliance 90 ml/cmH_2_O, resistance 15 cmH_2_O/l/s) at low (2.5 cmH_2_O) and high inspiratory efforts (10 cmH_2_O) at a respiratory rate of 15 and 30 breaths per minute. BiVent = time-cycled pressure controlled switching between two continuous positive airway pressure levels; HF-BiPAP = high flow biphasic positive airway pressure; PEEP = positive end-expiratory pressure; PSV = pressure support ventilation.

The pressure drop below PEEP (PTP_PEEP_) during unassisted breathing or at the lower Δ pressure is depicted in Figure 4. PTP_PEEP _was influenced by the Δ pressure (*P *< 0.001), the respiratory rate (*P *< 0.001), and the lung setting (*P *< 0.001). Overall mean PTP_PEEP _during HF-BiPAP was 0 ± 0.1 cmH_2_O/sec, compared with -0.13 ± 0.17 cmH_2_O/sec, and 0.23 ± 0.16 cmH_2_O/sec during BiVent and PSV respectively (*P *< 0.001). Mean fraction of PTP_PEEP _on PTPinsp accounted for 1.1 ± 3.3% (median 0%, 0/0.14%) during HF-BiPAP, while it was 3.4 ± 5.8% (median 0%, 0/4.8%) and 13.3 ± 30.9% (median 5.2%, 2.9/8.3%) during BiVent and PSV, respectively. In particular, at low inspiratory efforts the high flow during HF-BiPulse almost completely compensated the pressure drop, except for the normal lung condition. Even at high asynchrony setting (i.e. HF-BiPAP and BiVent at 15 breaths per minute, lung model rate 30 breaths per minute) the percentage of PTP_PEEP _in PTPinsp was lower during HF-BiPAP (mean 12.2 ± 46.8%, median 0%, 0/5.9%) when compared with PSV at 30 breaths per minute (mean 21.3 ± 41.9%, median 5.4%, 3.4/19.8%), but increased during BiVent (mean 30.1 ± 92%, median 0%, 0/10%).

**Figure 5:**
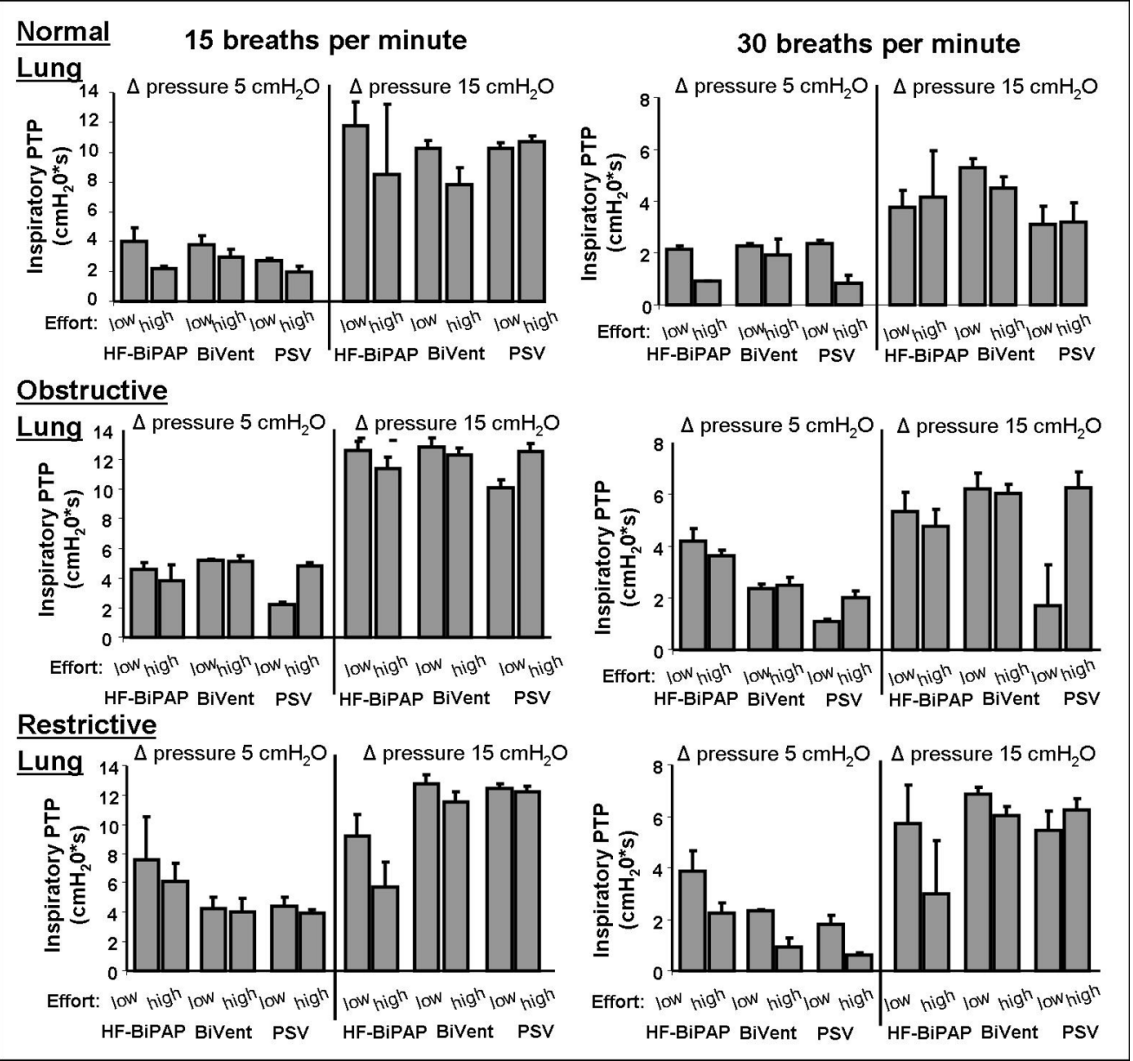
Effect of different ventilator respiratory settings on the mean inspiratory airway pressure time product (PTPinsp) during HF-BiPAP, BiVent, and PSV ventilation. Data were measured during normal (compliance 90 ml/cmH_2_O, resistance 3 cmH_2_O/l/s), restrictive (compliance 30 ml/cmH_2_O, resistance 3 cmH_2_O/l/s), and obstructive lung conditions (compliance 90 ml/cmH_2_O, resistance 15 cmH_2_O/l/s) at low (2.5 cmH_2_O) and high inspiratory efforts (10 cmH_2_O) at a respiratory rate of 15 and 30 breaths per minute. BiVent = time-cycled pressure controlled switching between two continuous positive airway pressure levels; HF-BiPAP = high flow biphasic positive airway pressure; PSV = pressure support ventilation.

Pressurization during inspiration is depicted in Figure 5 and Table 2. The values are reflected by the inspiratory pressure time products (PTPinsp). PTPinsp was influenced by the Δ pressure (*P *< 0.001), the respiratory rate (*P *< 0.001), and the lung setting (*P *< 0.001). Overall, PTPinsp differed significantly between the three ventilatory modes (*P *< 0.001). It tended towards higher PTPinsp during HF-BiPAP and BiVent compared with PSV (Figure 5). The HF-BiPAP system was more effective (HF-BiPAP 2.8 ± 1.2 cmH_2_O/sec, BiVent 2.1 ± 0.6 cmH_2_O/sec, PSV 1.5 ± 0.7 cmH_2_O/sec, *P *< 0.001), especially at a low Δ pressure and high respiratory rate. At a high respiratory rate of 30 breaths per minute with a HF-BiPAP/BiVent fixed at a cycling frequency of 15 breaths/minute minute, the time cycled modes were less effective (Table 2) if compared with a more synchronized breathing frequency (Table 1; HF-BiPAP *P *< 0.001, BiVent *P *< 0.001). If compared with PSV at 30 breaths per minute (2.9 ± 2.1), highly unsynchronized respiratory rates during HF-BiPulse (2.3 ± 2.1)/BiVent (2.4 ± 2.2) significantly differed (*P *= 0.0026) with regard to inspiratory pressurization.

**Table 2:** Mean inspiratory airway pressure (Paw mean), expiratory (PTPexp), inspiratory (PTPinsp) and PEEP (PTP_PEEP_) pressure time products at a respiratory rate of 30 breaths per minute and a ventilatory rate of 15 breaths per minute

**Δ pressure**			**5 **cmH_2_0		**15 **cmH_2_0	
**Effort**			low	high	low	high
**Normal lung condition**	**Paw mean **(cmH_2_O)	**HF-BiPAP nBBIPAPBiPulse**	10.5 ± 0.9	12.1 ± 0.2	14.6 ± 2.9	15.6 ± 2.4
		**BiVent**	10.6 ± 1.2	11.8 ± 1.8	15.3 ± 2.8	15.5 ± 2.2
	**PTP_PEEP_**. (cmH_2_0/sec)	**HF-BiPAP**	-0.1 ± 0.1	-0.2 ± 0.2	-0.2 ± 0.1	-0.1 ± 0.1
		**BiVent**	0 ± 0	-0.2 ± 0.1	0 ± 0	-0.3 ± 0.2
	**PTP insp**. (cmH_2_0/sec)	**HF-BiPAP**	1.2 ± 0.8	1.1 ± 0.7	3.3 ± 2.6	4 ± 1.7
		**BiVent**	1.3 ± 1.2	1.8 ± 1.5	2.7 ± 1.7	3.5 ± 2.4
	**PTP exp**. (cmH_2_0/sec)	**HF-BiPAP e**	7 ± 6.1	3.8 ± 1.8	10 ± 6.4	4.2 ± 1.8
		**BiVent**	7.2 ± 3.2	7.0 ± 0.9	11.8 ± 3.9	4.6 ± 1.8

**Obstructive lung condition**	**Paw mean **(cmH_2_O)	**HF-BiPAP**	10.4 ± 1	10.4 ± 0.7	15 ± 3.8	16.3 ± 3.4
		**BiVent**	10.3 ± 2.3	11 ± 3.5	15.4 ± 7.2	15.4 ± 4.1
	**PTP_PEEP_**. (cmH_2_0/sec)	**HF-BiPAP**	0 ± 0	-0.13 ± 0.15	-0.01 ± 0.05	-0.05 ± 0.16
		**BiVent**	0 ± 0	-0.2 ± 0.01	-0.02 ± 0.04	-0.2 ± 0.24
	**PTP insp**. (cmH_2_0/sec)	**HF-BiPAP**	1.0 ± 0.9	2.04 ± 1.4	3.6 ± 2.6	3.6 ± 2
		**BiVent**	1.3 ± 1.1	2.5 ± 2.4	2.8 ± 2.1	3.1 ± 2.7
	**PTP exp**. (cmH_2_0/sec)	**HF-BiPAP**	3.8 ± 2.5	9.8 ± 4.8	2.9 ± 1.7	10.1 ± 8.1
		**BiVent**	7.2 ± 3.2	10.9 ± 4.1	3.8 ± 1.9	10.3 ± 3.8

**Restrictive lung condition**	**Paw mean **(cmH_2_O)	**HF-BiPAP**	10.5 ± 1	11.7 ± 0.1	14.7 ± 2.2	14.7 ± 2.3
		**BiVent**	9.9 ± 1	11.1 ± 1	15 ± 2.8	15.4 ± 4.7
	**PTP_PEEP_**. (cmH_2_0/sec)	**HF-BiPAP**	-0.16 ± 0.2	-0.1 ± 0.2	0.09 ± 0.1	-0.09 ± 0.1
		**BiVent**	-0.35 ± 0.4	-0.24 ± 0.2	-0.16 ± 0.2	-0.16 ± 0.2
	**PTP insp**. (cmH_2_0/sec)	**HF-BiPAP**	1 ± 0.6	1.2 ± 0.2	3.3 ± 2	3.4 ± 2
		**BiVent**	2 ± 1.5	1.4 ± 0.9	3.3 ± 2.9	1.9 ± 2.4
	**PTP exp**. (cmH_2_0/sec)	**HF-BiPAP**	3.7 ± 1.7	7.8 ± 3.7	4.2 ± 2.4	9.4 ± 4.5
		**BiVent**	5 ± 1	7.2 ± 4.9	7.5 ± 3.2	10.1 ± 5.5

Measurements were made in normal (compliance 90 ml/cmH_2_O, resistance 3 cmH_2_O/l/s), obstructive (compliance 90 ml/cmH_2_O, resistance 15 cmH_2_O/l/s) and restrictive (compliance 30 ml/cmH_2_O, resistance 3 cmH_2_O/l/s) lung conditions, during varying delta pressure (Δ 5 and Δ 15 cmH_2_O) and inspiratory efforts (low: 2.5 cmH_2_O, high: 10 cmH_2_O) at a set positive end-expiratory pressure of 8 cmH_2_O.

HF-BiPAP = high flow biphasic positive airway pressure ventilation; BiVent = biphasic positive airway pressure delivered by a ventilator; PSV = pressure support ventilation by a ventilator.

The results of the PTPexp are summarised in Tables 1 and 2. Although PTPexp for the PEEP setting was subtracted, the ideal PTP should be zero. Thus, all the different ventilatory modalities led to an increase in pressurization beyond the expected PTPexp (*P *< 0.001). However, HF-BiPAP and BiVent PTPexp were higher than PSV (9.8 ± 5.8 cmH_2_O/sec vs. 8.8 ± 5.5 cmH_2_O/sec vs. 3.7 ± 1.9 cmH_2_O/sec; *P *< 0.001). As shown in Table 1, the level of the Δ pressure (*P *< 0.001), the lung condition (*P *= 0.001), respiratory rate (*P *< 0.001), and effort (*P *= 0.0015) also had significant effects on PTPexp. Asynchronous respiratory during biphasic pressure control did not led to a change in mean PTPexp during BiVent (*P *= 0.0998), while it was lower during HF-BiPAP (*P *= 0.001; Tables 1 and 2).

### Helmet and lung simulator ventilation

**Figure 6:**
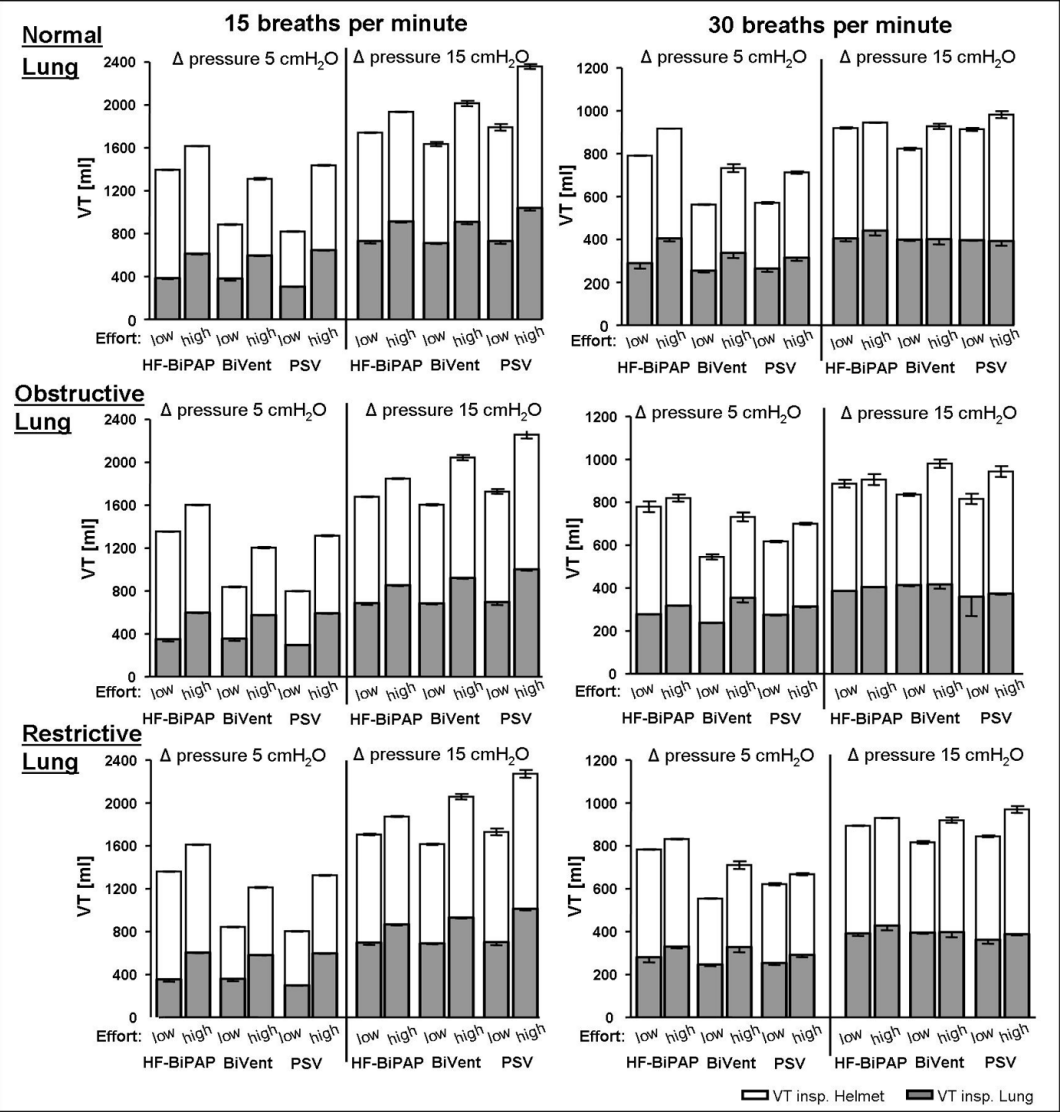
Effect of different ventilator respiratory settings and respiratory rate on mean tidal volume delivered to the helmet (VTinsp Helmet) and to the Active Simulator Lung (VTinsp Lung) with HF-BiPAP, BiVent, and PSV ventilation. Measurements were made in normal (compliance 90 ml/cmH_2_O, resistance 3 cmH_2_O/l/s), restrictive (compliance 30 ml/cmH_2_O, resistance 3 cmH_2_O/l/s), and obstructive lung conditions (compliance 90 ml/cmH_2_O, resistance 15 cmH_2_O/l/s) at low (2.5 cmH_2_O) and high inspiratory efforts (10 cmH_2_O) at a respiratory rate of 15 and 30 breaths per minute. Grey columns = inspiratory VT to the Active Lung Simulator (VTinsp Lung); white columns = inspiratory VT to the Helmet (VTinsp helmet). BiVent = time-cycled pressure controlled switching between two continuous positive airway pressure levels; HF-BiPAP = high flow biphasic positive airway pressure; PSV = pressure support ventilation.

Tidal volumes delivered to the helmet and the lung simulator are depicted in Figure 6. Tidal volumes delivered to the helmet differed markedly to those delivered to the lung (*P *< 0.001). Only about 75% of the ventilatory tidal volume reached the lung. Tidal volumes to the helmet were higher during HF-BiPAP (753 ± 250 ml) than BiVent (604 ± 264 ml) and PSV (669 ± 312 ml; *P *< 0.001). Overall, there was no significant difference in the tidal volumes delivered to the lung model (HF-BiPAP: 502 ± 196 ml, BiVent: 476 ± 176 ml PSV: 424 ± 173; *P *= 0.932). However, the HF-BiPAP system was more effective (HF-BiPAP 318 ± 48 ml, BiVent 294 ± 51 ml, PSV 286 ± 26 ml, *P *< 0.001) at a low Δ pressure and a high respiratory rate.

### Carbon dioxide removal

The CO_2 _elimination was significantly influenced by the ventilator mode (*P *< 0.001), the height of the Δ pressure (*P *< 0.001) and the lung condition (*P *= 0.018; Table 1). The HF-BiPAP system had a mean maximum and minimum CO_2 _concentration of 0.15 ± 0.1% and 0.02 ± 0.2% respectively. In contrast, the PSV system had respective values of 0.54 ± 0.3% and 0.19 ± 0.1% and the BiVent system had respective values of 3.3 ± 1.1% and 0.37 ± 0.2%. Maximum CO_2 _during HF-BiPAP was only influenced by the height of the Δ pressure (5 cmH_2_O: 0.11 ± 0.02%, 15 cmH_2_O: 0.19 ± 0.1%; *P *< 0.001). During PSV it was significantly changed by the Δ pressure, effort (*P *= 0.015) and the lung condition (*P *= 0.001). Thus the HF-BiPAP assured low CO_2 _concentrations at all settings. During BiVent, the maximum CO_2 _concentrations were particularly high.

### Noise exposure

Mean ambient noise level without activation of the ventilator was 43.7 ± 0.1 dBA. It increased to 45.5 ± 1.8 dBA when the simulator was activated. Respiratory rate and lung settings influenced the baseline noise level. The level ranged between 44.1 ± 0.4 dBA (15 bpm, low effort and normal lung condition) and 49.4 ± 1.7 dBA (30 bpm, high effort, normal lung condition) during the different settings. The mean noise level during HF-BiPAP, PSV, and BiVent were 72.2 ± 5.7 dBA (min: 60.4 dBA, max: 84 dBA), 60.2 ± 5 dBA (min: 49.6 dBA, max: 73.4 dBA), and 59.8 ± 5.4 dBA (min: 49.3 dBA, max: 71.2 dBA), respectively (*P *< 0.0001).

## Discussion

The present *in vitro *study demonstrates that HF-BiPAP: provides adequate pressurization during inspiration especially at lower levels of assistance in both restrictive and obstructive lung conditions even when there is high inspiratory effort; compensates a pressure drop below the lower pressure level during inspiration more efficiently than time-cycled pressure control or PSV; delivers tidal volumes that are overall comparable with BiVent or PSV, but is more efficient at lower levels of assistance and high respiratory rates; is highly efficient at CO_2 _washout regardless of the effort and clinical condition; and during high asynchrony (ventilator rate at 15 breaths per minute with a inspiratory:expiratory ratio of 1:1 at a respiratory rate of 30 breaths per minute) both HF-BiPAP and BiVent are less efficient.

Previous studies comparing the helmet to the facemask interface have shown that the helmet is less effective at unloading the respiratory muscles, due to inspiratory trigger delays and impaired pressurization rate [17-19]. Possible solutions include changing the mode of triggering [22,23] or using a mode that does not rely on triggering at all. One such mode would be a time-cycled biphasic positive pressure ventilation.

In our study, we compared the efficiency of BiVent and PSV. Among the clinical conditions tested, we found an increase in the mean airway pressure during BiVent. This can be explained by the fact that the time-cycled mode of BiVent, results in a fixed inspiratory time. Meanwhile, a flow-cycled mode, such as PSV, results in variable inspiratory time [24]. The PTP_PEEP _was lower in BiVent compared with PSV during the general settings. This may be explained by the fact that breaths can be initiated at the upper pressure level without any trigger delay or pressure drop below PEEP. However, during very asynchronous BiVent, PTP_PEEP _was worse compared with the PSV setting. Furthermore, the inspiratory pressurization (PTPinsp) tended to be higher in BiVent than PSV, although this was not the case during high asynchrony. On the other hand, the PTPexp was increased. This increase was independent of the effort and the clinical condition. This finding may be due to impaired synchrony. Unlike conventional PSV, BiVent does not adjust to the patient's inspiratory and expiratory cycles. Although the increased PTPexp might add an additional burden by increasing the expiratory work of breathing it might also have some beneficial effects during BiVent, where a higher pressure during the expiratory pause may allow an increase of lung volume and possibility of performing CPAP at the higher pressure level during the next breath.

Efficient CO_2 _removal from the respiratory circuit is a mandatory requirement for NIV optimisation. Removal might be a problem when using the helmet with a mechanical ventilator [20]. The maximum and minimum CO_2 _concentrations were higher with BiVent than PSV. Increasing the level of assistance resulted in a minimal reduction in CO_2 _in both ventilatory modes. The less efficient CO_2 _washout during BiVent could be attributed to the unsynchronized cycling, mainly in the expiratory phase in the presence of a relatively low bias flow from the ventilator. The level of clinically relevant inspiratory CO_2 _during NIV has not been defined. However, depending on the inner volume and location of the exhalation port on the mask, CO_2 _rebreathing can also be seen with other interfaces such as conventional face masks [16,25,26]. During BiVent the maximum helmet CO_2 _concentrations ranged between 1.1 and 4.9%. These findings might demonstrate a risk in hypercapnic patients requiring NIV.

Compared with BiVent, HF-BiPAP is characterized by a high free flow and the fact that pressurization within the interface is caused by a time-cycled expiratory resistor.

The helmet has failed to replace the mask as a commonly used interface during non-invasive positive pressure ventilation because of poor patient-ventilator interaction. Poor interaction leads to inefficient muscle loading, increased work of breathing [17], and reduced CO_2 _washout. It has been suggested that high flows are indicated when CPAP is applied via the helmet [16,20,27]. However, CPAP does not provide efficient respiratory assistance [28]. The main advantages of HF-BiPAP are that it combines the positive effects of high free flow, minimizes CO_2 _rebreathing, and has adjustable inspiratory assistance.

Our data shows that the HF-BiPAP system is capable of efficiently pressurizing the helmet while keeping the set lower pressure level. HF-BiPAP worked well in the presence of all lung conditions and respiratory rates. The direct effect of a high flow on pressurization is shown by the compensated negative pressure drop during inspiration at the lower pressure level in almost all settings. Even with high asynchrony, PTP_PEEP _was less affected compared with BiVent or conventional PSV. Many studies regarding the helmet interface focused on inspiratory delay and its effect on pressurization, so this finding is of major interest because poor synchrony can be partially compensated by a high flow allowing stable pressurization.

A major critique of our approach is that asynchrony in this setting can be worse than in PSV. This assumption is reflected by the finding that PTPinsp was less effective during asynchronous HF-BiPAP/BiVent at a ventilator rate of 15 breaths per minute and a respiratory rate of 30 breaths per minute. Based on these findings an adjustment of the ventilator cycling close to the patient's respiratory rate would be advisable, although this might not completely resolve the problem. In fact desynchronization may occur solely on the lower pressure level with no adjustment to the patient's inspiratory and expiratory cycling. However, the patient can spontaneously breathe in the helmet, while receiving adequate flow during inspiration. During this mode of helmet NIV, synchronization might be less important compared with conventional interfaces of non-invasive ventilation.

The efficiency of the inspiratory pressurization was altered by the clinical condition and inspiratory effort. In normal and obstructive conditions, HF-BiPAP was as efficient as PSV and BiVent. In restrictive conditions, the device became less efficient at higher inspiratory effort and level of assistance. On the other hand, we observed an increase in expiratory load in all clinical conditions. This was likely to be because of asynchrony in the expiratory phase and the continuous high flow in the circuit probably resulting in an increase of the expiratory resistance. Overall, this resulted in an increase in mean airway pressure. On the other hand, the tidal volumes delivered to the lungs were comparable with BiVent and PSV. The volumes delivered were even more efficient at lower levels of assistance and higher respiratory rates.

In our *in vitro *study the use of a HF-BiPAP system led to efficient CO_2 _removal. This removal occurred regardless of the underlying lung pathology. These effects were not caused by the ventilatory mode as shown by the negative effect of conventional times cycled biphasic pressure ventilation (BiVent) but by the high continuous flow [20]. Thus, an adjustable bias flow would eliminate the potential problem of CO_2 _rebreathing in any conventional ventilatory mode of NIV via the helmet interface.

The mean noise exposure imposed by the HF-BiPAP was higher than that of PSV and BiVent. The higher mean noise was caused by both the higher average but also the higher peak noise level. Cavaliere and colleagues described a noise level of 94 ± 2 dBA when using the helmet with a CPAP system compared with 57 ± 11 dBA for a mechanical ventilator plus mask [29]. We measured the ambient noise level in our intensive care unit next to a patient who was not mechanical ventilated. The mean noise level over two hours ranged between 40 and 75 dBA depending on the time of the day, alarms, and activities at the bedside. Thus the additional noise exposure caused by the ventilator is rather overestimated in the laboratory conditions. A proper noise damping of the prototype might improve noise exposure.

Our study had several limitations that need to be addressed in order to properly interpret the results.

First, we used a lung model with a fixed inspiratory time. This time was unaffected by the level of respiratory assistance. Fixed combinations of compliance and resistance were used to determine the different clinical conditions. Moreover, the model passively reacted to the different levels and modalities of assistance. The results of the study are sensitive to the experimental setting, and choosing respiratory rates different to those tested in the study could have given different results. Therefore, our data must be considered a preliminary attempt to evaluate the interaction between different ventilatory modalities and the helmet, and need to be verified in a clinical study.

Second, in actual patients, the source of asynchrony in the helmet might be a variable respiratory pattern. In the lung model tested, respiratory patterns were fixed, even though different mechanical settings of the lung model were used. This approach increased the risk for asynchrony. The increased risk might outweigh the benefits of using fixed settings to represent the respiratory pattern.

Third, we did not evaluate the effects of the different systems on the work of breathing. This lack of evaluation was due to the limitations of our model. Instead, we used the changes in pressurization at different inspiratory efforts to evaluate the efficiency of the different devices.

Fourth, different ventilatory modalities were tested during ideal conditions (no leakage). Inspiratory triggering and end inspiratory cycling off are particularly sensitive to air leaks. Thus the comparable efficiency of PSV was rather overestimated. In practice, more leaks are to be expected. PSV would result in higher inspiratory and expiratory delays. These delays would result in a lower inspiratory PTP than the time-cycled modalities.

Fifth, the CO_2 _model has some limitations. The artificial lung model is not actively involved in gas exchange. Therefore a change in tidal volume, minute ventilation, or underlying lung condition did not directly affect the CO_2 _load to the helmet. Interpretations of the impact of the lung pathology on the CO_2 _concentration within the helmet should be drawn with caution.

Finally, we only examined the helmet. For this reason, we cannot directly extrapolate our results to other interfaces including nasal mask, facial masks, or endotracheal tubes.

## Conclusions

This study demonstrates a new concept of time-cycled biphasic airway pressure with a high flow system in a lung model. This approach might resolve some of the problems seen with the helmet interface and broaden the indications for its use. With HF-BiPAP, CO_2 _removal from the helmet was highly efficient regardless of the underlying lung pathology. Pressurization of the helmet seems particularly efficient at the lower levels of assistance. If the patient requires a high level of assistance one has to be aware of the increased pressurization during expiration, which might be beneficial but also increases the load during expiration.

## Key messages

• HF-BiPAP provides adequate pressurization at low levels of assistance. It is effective in both restrictive and obstructive lung conditions, even when there is high inspiratory effort.

• HF-BiPAP compensates a pressure drop below the lower pressure level during inspiration more efficiently than time-cycled pressure control or PSV.

• HF-BiPAP delivers tidal volumes that are comparable with BiVent or PSV. These volumes are even more efficient at low levels of assistance and higher respiratory rates.

• HF-BiPAP ensures an efficient CO_2 _washout, regardless on the effort and clinical condition.

## Abbreviations

ANOVA: analysis of variance; BiVent: time-cycled pressure controlled switching between two continuous positive airway pressure levels; CO_2_: carbon dioxide; CPAP: continuous positive airway pressure; HF-BiPAP: high flow biphasic positive airway pressure; NIV: non-invasive ventilation; PEEP: positive end-expiratory pressure; PSV: pressure support ventilation; PTP: pressure time product.

## Competing interests

The authors declare that they have no competing interests.

## Authors' contributions

OM, PH, MQ, and PP planed and designed the study and developed the measurement setup. OM, PH, and PP performed the measurements and analyzed the data. PS and EC performed pilot studies prior to the project and helped analyzing and drafting the manuscript as well as JH. All authors (OM, PH, JH, PS, EC, MQ, and PP) participated in the analysis and interpretation of the results. The final manuscript was written by OM in close cooperation with PP, assisted by all participating authors (PH, JH, PS, EC, and MQ) who helped to draft and revise the manuscript.
